# The epidemiology of cubital tunnel syndrome: a UK Biobank case–control study

**DOI:** 10.1177/17531934261426700

**Published:** 2026-03-11

**Authors:** Maria Lucey, Frederik Heymann Lassen, Dominic Furniss, Akira Wiberg

**Affiliations:** 1 Botnar Research Centre, Nuffield Department of Orthopaedics, Rheumatology, and Musculoskeletal Science, University of Oxford, UK; 2 Centre for Human Genetics, University of Oxford, UK; 3 Big Data Institute, Li Ka Shing Centre for Health Information and Discovery, University of Oxford, UK; 4 Department of Plastic and Reconstructive Surgery, Oxford University Hospitals NHS Foundation Trust, John Radcliffe Hospital, UK

**Keywords:** Biobank, cubital tunnel syndrome, epidemiology, risk factors, modifiable, primary prevention, secondary prevention

## Abstract

**Introduction:**

Cubital tunnel syndrome is the second most prevalent upper limb compression neuropathy. Limitations in non-operative and surgical treatment warrant an adjunctive preventative approach targeting modifiable risk factors. However, risk factors are poorly characterized given methodologic shortcomings in previous studies.

**Methods:**

In this case–control study in the UK Biobank (1971 cases, 398,639 controls), multivariable logistic regression with extensive covariate correction was conducted to assess the association between cubital tunnel syndrome and sex, age, ethnicity, socioeconomic deprivation, smoking status, alcohol intake, height, body mass index, plasma lipids, HbA1c, diabetes, hypertension, carpal tunnel syndrome, osteoarthritis, rheumatoid arthritis, hypothyroidism and gastroesophageal reflux disease.

**Results:**

Cubital tunnel syndrome was significantly associated with several modifiable risk factors: smoking status (odds ratio 2.08, 95% CI: 1.83–2.37), diabetes with microvascular complications (OR 1.80, 95% CI: 1.40–2.30), hypertension (OR 1.37, 95% CI: 1.24–1.52) and raised body mass index (OR 1.13, 95% CI: 1.08–1.19). Male sex (OR 1.77, 95% CI: 1.54–2.04) and age (OR 0.981, 95% CI: 0.975–0.986) were non-modifiable risk factors. Several comorbidities were associated with cubital tunnel syndrome: carpal tunnel syndrome (OR 11.7, 95% CI: 10.6–12.9), osteoarthritis (OR 2.12, 95% CI: 1.92–2.34), rheumatoid arthritis (OR 1.72, 95% CI: 1.42–2.07), and gastroesophageal reflux disease (OR 1.66, 95% CI: 1.50–1.83). Cubital tunnel syndrome had a weak association with socioeconomic deprivation and no association with ethnicity, height, alcohol intake, plasma lipids, HbA1c and hypothyroidism.

**Conclusion:**

Cubital tunnel syndrome is robustly associated with several modifiable risk factors that may be targeted in primary and secondary prevention.

**Level of evidence:**

III

## Introduction

Ulnar nerve compression at the elbow, known as cubital tunnel syndrome (CuTS), is the second most common upper limb compression neuropathy, with a prevalence of 1.8–5.9% (An et al., 2017). Surgical intervention for CuTS has risen steadily (Bebbington and Furniss, 2015); despite this, there is little consensus on the optimal surgical approach (Burahee et al., 2022; Graf et al., 2023) and revision rates owing to symptom recurrence are high, ranging from 2 to 25% (Hutchinson et al., 2021; Wade et al., 2020). While mild to moderate cases may be treated with non-operative strategies (Dellon et al., 1993; Graf et al., 2023), including activity modification, nocturnal splinting, and nerve gliding exercises, inconsistent reports of efficacy have led to a lack of standardized non-operative treatment (Graf et al., 2023; Shah et al., 2013; Svernlöv et al., 2009). The existing treatment arsenal to prevent disease progression is thus far from ideal, warranting identification of modifiable risk factors for primary or secondary prevention.

Previous studies on CuTS risk factors have been limited by small sample sizes, hospital-based cohorts, the use of self-reported diagnoses and minimal confounder adjustment, leading to low statistical power, poor generalizability, type I error and inaccurate effect size estimates. This has generated considerable uncertainty in the literature on CuTS, with inconsistent evidence for associations with older age (Grisdela Jr et al., 2025; Rocks et al., 2024), male sex (Bartels and Verbeek, 2007; Mondelli et al., 2020), socioeconomic deprivation (Grisdela Jr et al., 2025; Johnson et al., 2021a; Zimmerman et al., 2020), increased body mass index (BMI) (Bartels and Verbeek, 2007; Mondelli et al., 2020), alcohol intake (Rocks et al., 2024; Rydberg et al., 2020) and diabetes (Rocks et al., 2024; Rydberg et al., 2020). Weak evidence exists for associations with hypothyroidism (Miettinen et al., 2021) and rheumatoid arthritis (Rocks et al., 2024). The only consistently associated exposures are smoking (Bartels and Verbeek, 2007; Frost et al., 2013; Mondelli et al., 2020; Richardson et al., 2009) and carpal tunnel syndrome (Johnson et al., 2021b; Shulman et al., 2020).

We aimed to overcome the methodologic challenges of previous studies by: (a) studying the large and deeply phenotyped, population-based UK Biobank cohort; (b) using a strict definition of CuTS based on primary and secondary care diagnostic codes; and (c) applying multivariable fixed effects logistic regression to accurately determine the degree of association between CuTS and various exposures.

## Methods

### Study design and participants

We designed a case–control study within the UK Biobank (UKB) cohort, a prospective, population-based national cohort of 502,128 participants, recruited between 2006 and 2010 across 21 assessment centres in England, Scotland and Wales. Inclusion criteria were: being registered with the NHS; living within 25 miles of one of the 22 test centres; and being aged 40–69 years. The UKB integrates primary and secondary healthcare data, combining hospital and GP records, self-reported health and lifestyle data, blood biochemistry, anthropometric measurements, genetic data, and imaging data. The Biobank is regularly updated with data on new diagnoses, with the last update in 2025. All participants provided informed consent for the inclusion of their data.

### Case definition

Cubital tunnel syndrome cases were identified using: Office of Population Censuses and Surveys (OPCS) Classification of Interventions and Procedures, version 4 (OPCS-4) codes; International Classification of Diseases, 10th Revision (ICD-10) and 9th Revision (ICD-9) codes; and Read Codes (Clinical Terms Version 3 (CTV3)) (Tables S1 and S2). The following were included, yielding 2487 cases:

1. OPCS-4: A67.1 ‘Cubital tunnel release’;
2. ICD-10: G56.2 ‘Lesions of ulnar nerve’;
3. ICD-9: 3542 ‘Lesions of ulnar nerve’; and
4. CTV3: F3420 ‘Cubital tunnel syndrome’, 70580 ‘Decompression of ulnar nerve at elbow’, X00Br ‘Ulnar nerve entrapment at elbow’.

Sensitivity analysis was conducted using a more restrictive definition of CuTS that included the above but combined ICD-10 and ICD-9 codes with OPCS-4 Z81.5 ‘Elbow joint’, yielding 1829 cases (Table S3).

### Phenotypic association analysis

To identify diagnoses associated with CuTS, three-digit ICD-10 code phenotypes with more than 500 cases in the UKB were systematically examined (1100 phenotypes). Two-by-two contingency tables were constructed for each CuTS-ICD-10-phenotype pair and a one-way Fisher’s exact test was conducted. Results were plotted as −log10(*p*-value) for each phenotype, grouped by ICD-10 chapter. Significance threshold was set at Bonferroni-corrected *p* = 4.55 × 10^−5^ (0.05/1100 tests).

### Selection of exposure variables

Exposures studied were age, sex, ethnicity, Townsend deprivation index (a measure of socioeconomic deprivation), smoking status, alcohol intake, height, BMI, plasma lipids (total cholesterol, triglycerides, low-density lipoprotein (LDL) and high-density lipoprotein (HDL)), glycated haemoglobin (HbA1c) and selected comorbidities (diabetes, hypertension, carpal tunnel syndrome (CTS), osteoarthritis, rheumatoid arthritis (RA), hypothyroidism, and gastroesophageal reflux disease (GORD)). Exposure variables were selected on the basis of a literature review and phenotypic association analysis. Literature review supported the inclusion of age, sex, BMI, socioeconomic deprivation, smoking, alcohol intake, diabetes, hypothyroidism, RA and CTS. Height was included as a proxy for cubital tunnel anatomical differences (Lee et al., 2020). The phenotypic association study supported the inclusion of hypertension, hypercholesterolaemia (plasma lipids as a proxy), obesity (BMI as a proxy), osteoarthritis and GORD.

### Exposure measurement

Age was taken at date of recruitment to UKB. Sex and ethnicity were self-reported. Townsend deprivation index was calculated at the time of recruitment to UKB based on national census data linked to the participant’s postcode (more positive values denote increased socioeconomic deprivation). Smoking status was self-reported as ‘never’, ‘previous’, ‘current’ or ‘prefer not to answer’. Alcohol intake was reported as ‘daily or almost daily’, ‘three to four times a week’, ‘once or twice a week’, ‘one to three times a month’, ‘special occasions only’, ‘never’ or ‘prefer not to answer’. Individuals who answered ‘prefer not to answer’ or ‘do not know’ for ethnicity, smoking status and alcohol intake were excluded. Height and weight were measured by trained staff at recruitment; BMI was calculated from the measurements. the BMI categories were defined as: underweight <18.5, normal ⩾18.5–25, overweight ⩾25–30, and obese >30. Plasma lipids and HbA1c were measured at baseline and follow-up assessments. Smoking status, alcohol intake, and BMI categories were analysed as ordinal categorical variables.

Selected comorbidities were identified using OPCS-4, ICD-10, ICD-9 and CTV3 codes (Tables S1 and S2). Both type 1 and type 2 diabetes were included, and recategorized into diabetes with and without microvascular complications (neuropathy, nephropathy and retinopathy). Diabetes was analysed as an ordinal categorical variable (no diabetes, diabetes without complications and diabetes with complications).

### Statistical analysis

Descriptive statistics were used to compare baseline characteristics between cases and controls (Table 1). Missingness was present in some variables. A systematic analysis of missingness justified complete case analysis (Supplementary note 1, Tables S4 and S5). Using complete case analysis, the univariable and fully adjusted multivariable models had 400,610 participants: 398,639 controls and 1971 CuTS cases.

**Table 1.** Baseline descriptive statistics of cubital tunnel cases and controls. Baseline descriptive statistics of cubital tunnel cases and controls, with the number of missing individuals reported for each exposure.

Exposure	Cubital tunnel syndrome cases (%)	Controls (%)	Missing, *n* (%)	
			Cases	Controls
Total number	2487	499,641	–	–
Age at entry (years)			–	–
Median	59	58	–	–
IQR	12	13	–	–
Sex				
Female	1045 (42.0)	272,110 (54.5)	–	–
Male	1442 (58.0)	227,531 (45.5)	–	–
Ethnicity			15 (0.60)	2760 (0.55)
White	2,360 (94.9)	470,000 (94.1)	–	–
Afro-Caribbean	41 (1.65)	8007 (1.60)	–	–
Asian	43 (1.73)	11,400 (2.28)	–	–
Mixed	14 (0.56)	2936 (0.59)	–	–
Other	14 (0.56)	4538 (0.91)	–	–
Townsend deprivation index			2 (0.08)	622 (0.12)
Median	−1.49	−2.14	–	–
IQR	4.85	4.18	–	–
Smoking			12 (0.48)	2936 (0.59)
Never	998 (40.1)	272,325 (54.5)	–	–
Current	471 (18.9)	52,466 (10.5)	–	–
Previous	1006 (40.5)	171,914 (34.4)	–	–
Alcohol intake			6 (0.24)	1494 (0.30)
Never	272 (10.9)	40,323 (8.07)	–	–
Special occasions only	330 (13.3)	57,636 (11.5)	–	–
1 – 3 times per month	270 (10.9)	55,542 (11.1)	–	–
1 – 2 times per week	629 (25.3)	128,555 (25.7)	–	–
3 – 4 times per week	497 (20.0)	114,859 (23.0)	–	–
Daily or almost daily	483 (19.4)	101,232 (20.3)	–	–
Anthropometric factors				
Height (cm)			28 (1.13)	2509 (0.50)
Median	170	168	–	–
IQR	13	13.4	–	–
BMI (kg/m^2^)			31 (1.25)	3072 (0.61)
Median	28.4	26.7	–	–
IQR	6.52	5.77	–	–
Plasma lipids				
Mean total cholesterol ± SD (mmol/L)	5.59 ± 1.22	5.69 ± 1.15	165 (6.63)	32,720 (6.55)
Triglycerides (mmol/L)			168 (6.76)	33,092 (6.62)
Median	1.67	1.48	–	–
IQR	1.28	1.10	–	–
LDL (mmol/)			173 (6.69)	33,594 (6.72)
Median	3.45	3.52	–	–
IQR	1.25	1.17	–	–
HDL (mmol/L)			362 (14.6)	72,212 (14.5)
Median	1.31	1.40	–	–
IQR	0.50	0.50	–	–
Glycaemic factors				
HbA1c (mmol/mol)			172 (6.92)	35,794 (7.16)
Median	36.2	35.2	–	–
IQR	6.00	5.1	–	–
Diabetes mellitus without complications	404 (16.2)	42,512 (8.51)	–	–
Diabetes mellitus with complications	150 (6.03)	8,505 (1.70)	–	–
Co-morbidities				
Hypertension	1464 (58.9)	201,150 (40.3)	–	–
Carpal tunnel syndrome	964 (38.8)	23,334 (4.67)	–	–
Osteoarthritis	1270 (51.1)	122,509 (24.5)		
Rheumatoid arthritis	154 (6.19)	11,119 (2.23)	–	–
Hypothyroidism	259 (10.4)	39,757 (7.96)	–	–
GORD	738 (29.7)	77,991 (15.6)	–	–

IQR: interquartile range.

Logistic regression was performed to assess the association between exposures and CuTS. The first model was a univariable model estimating the association of each variable (no adjustments). The second was a fully adjusted multivariable model, adjusted for age, sex, ethnicity, Townsend deprivation index, height, BMI, triglycerides, LDL, HDL, HbA1c, alcohol, smoking, diabetes with and without complications, hypertension, CTS, RA, hypothyroidism and GORD. Total cholesterol was excluded owing to collinearity with HDL and LDL cholesterol. Osteoarthritis was excluded from the fully adjusted model because it was found to act as a mediator (Pearl, 2001) between age and CuTS. Thus, to estimate the total effect of age on CuTS risk (both direct and indirect pathways), we avoided adjusting for this intermediate variable that likely lies on the causal pathway.

A baseline reference was set for several variables: alcohol as ‘once or twice a week’; smoking as ‘never’; ethnicity as ‘white’; and sex as ‘female’. HbA1c was centred to 42 mmol/mol (upper end of the non-diabetic range) and scaled so that each unit increase in odds ratio (OR) corresponded to a 10 mmol/mol or 0.9% increase in HbA1c. Height was scaled by the standard deviation of the overall cohort (9.28 cm) and BMI was scaled by 5 kg/m^2^.

Given the exploratory nature of the analyses, a Bonferroni correction was applied to correct for multiple testing in the multivariable analysis, with the significance threshold set at *p* < 1.85 × 10^−3^ (0.05/27 tests).

## Results

Of the 502,128 participants, there were 2487 CuTS cases and 499,641 controls (Table 1).

### Phenotypic association analysis

**Figure 1.**
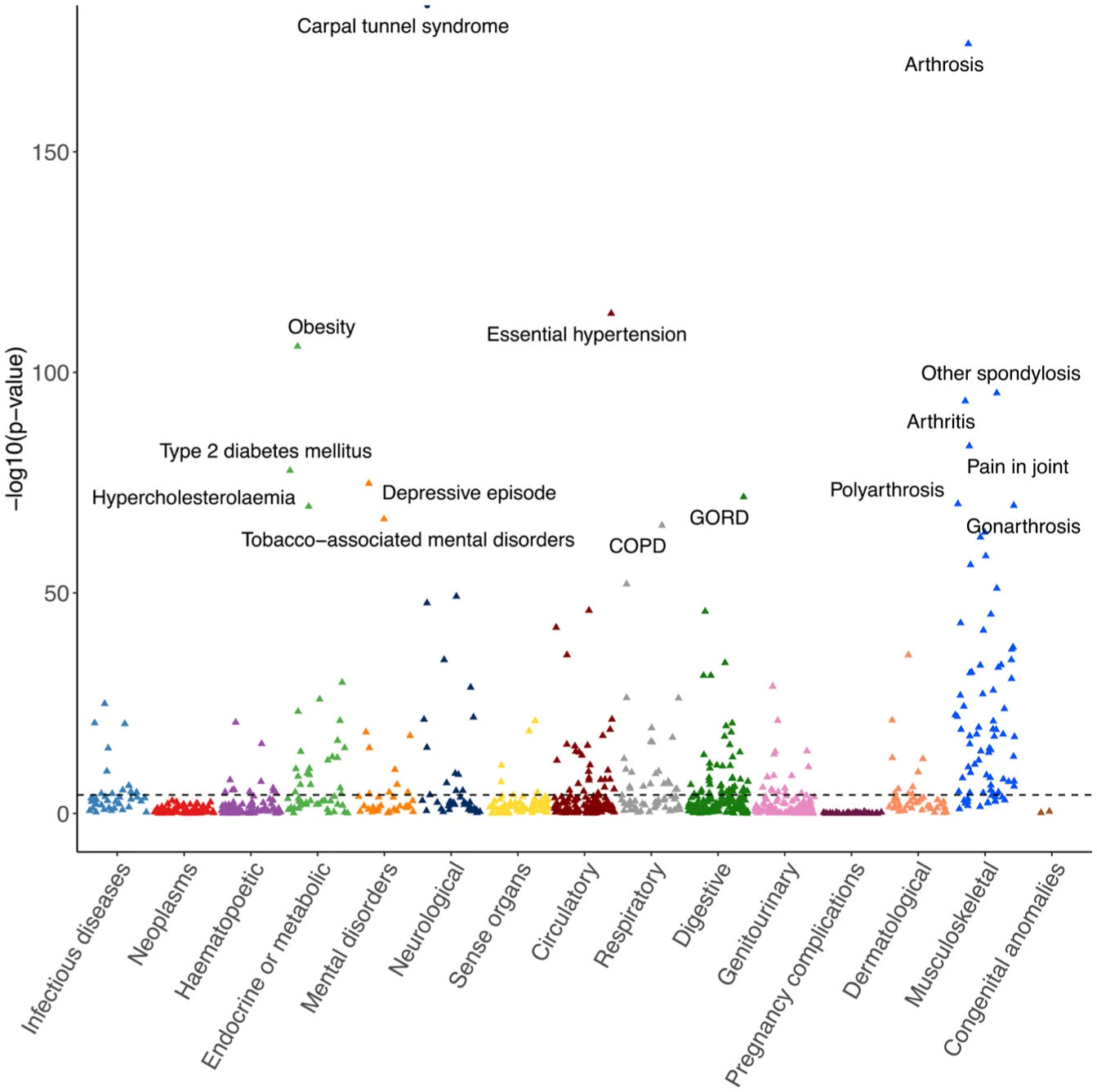
Phenotypic association analysis examining the association of cubital tunnel syndrome with 1100 phenotypes derived from ICD-10 coding in the UK Biobank, grouped by ICD-10 chapter. Highly significant associations were seen for carpal tunnel syndrome, osteoarthritis (arthrosis, arthritis, gonarthrosis), hypertension, obesity, type 2 diabetes mellitus, depression, GORD, hypercholesterolaemia and smoking-related diagnoses (COPD, tobacco-associated mental disorders). *P*-values were determined using one-way Fisher’s exact test. Diagnoses are labelled where *p* < 1 × 10^−65^. Carpal tunnel syndrome point is labelled as a circle to indicate that the *p*-value has been truncated for visualization purposes (*p* < 1 × 10^−175^, limit of *y*-axis). COPD: chronic obstructive pulmonary disease; GORD: gastroesophageal reflux disease.

Phenotypic association analysis was conducted as a screen for associations between CuTS and ICD-10-based phenotypes to help inform the selection of exposures for study in our regression models. Highly significant associations were observed for a number of phenotypes, including CTS (*p* < 2.23 × 10^−308^), osteoarthritis (*p* = 3.62 × 10^−175^), hypertension (*p* = 4.39 × 10^−114^), obesity (*p* = 1.28 × 10^−106^), type 2 diabetes (*p* = 1.80 × 10^−78^), hypercholesterolaemia (*p* = 2.45 × 10^−70^), GORD (*p* = 1.78 × 10^−72^) and two smoking-related diagnoses (chronic obstructive pulmonary disease, *p* = 4.98 × 10^−66^, and tobacco-related mental disorders, *p* = 1.81 × 10^−67^) (Figure 1; Table S6).

### Non-modifiable exposures

**Figure 2.**
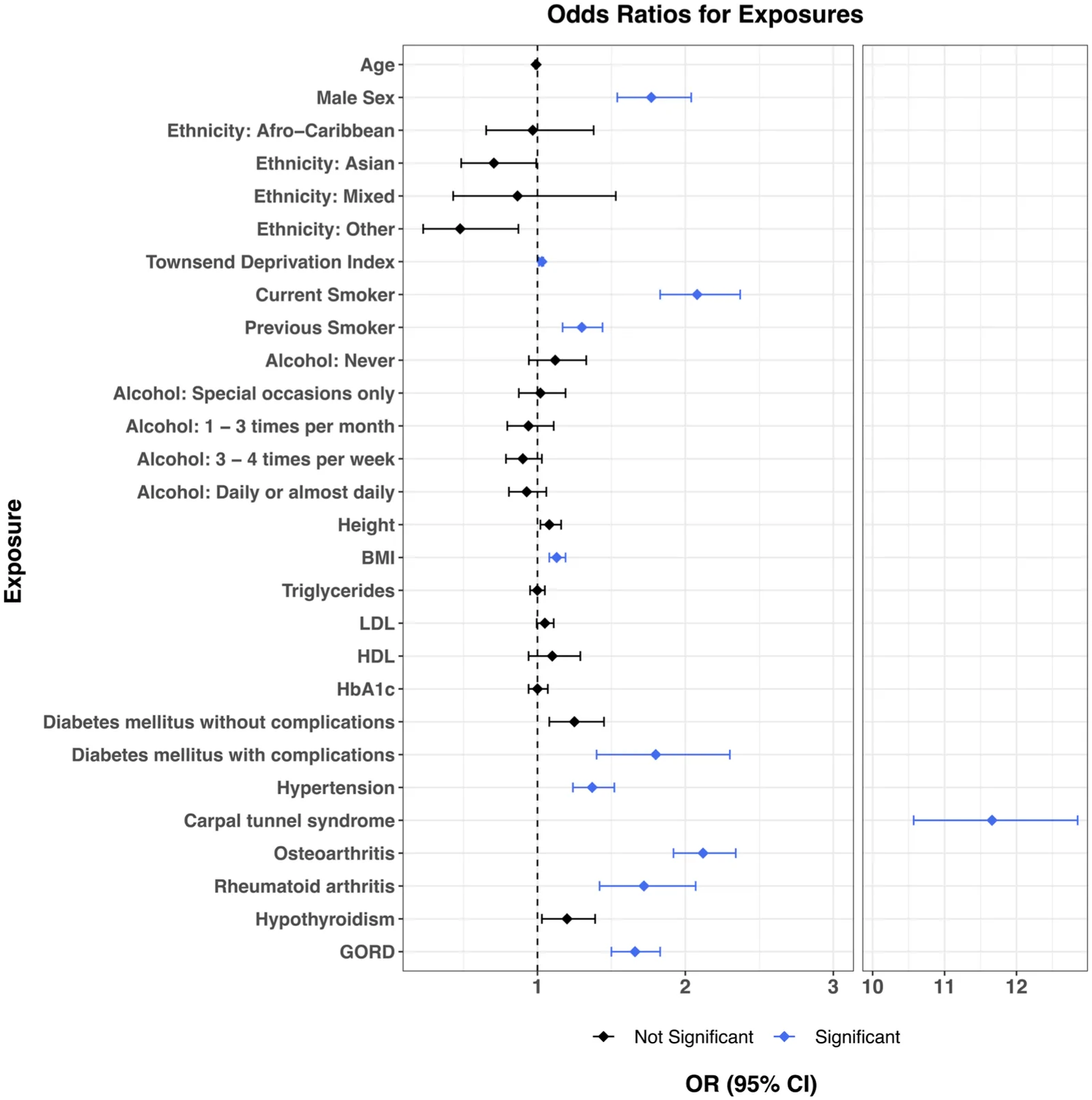
Forest plot of odds ratios. Odds ratios and 95% confidence intervals for the association of exposures with cubital tunnel syndrome. Significant associations shown in blue where *p* < 1.85 × 10^−3^. Odds ratios, 95% confidence intervals and *p*-values taken from the fully adjusted model.

Male sex was significantly associated with CuTS (Table 2, Figure 2). After full adjustment, males had 77% increased odds of CuTS compared with females (OR 1.77, 95% CI: 1.54–2.04, *p* = 1.50 × 10^−15^). There was no association between different ethnicities and CuTS.

**Table 2.** Univariable and fully adjusted logistic regression ORs for cubital tunnel syndrome.

Exposure	Univariable OR (95% CI)	*p*-value	Fully adjusted multivariable OR (95% CI)	*p*-value
Age	1.01 (1.00 – 1.01)	1.98 × 10^−3^	0.991 (0.984 – 0.997)	2.97 × 10^−3^
Age: with osteoarthritis	1.00 (0.996 – 1.01)	0.591	0.988 (0.982 – 0.994)	2.15 × 10^−4^
Age: no osteoarthritis	0.981 (0.975 – 0.987)	1.38 × 10^−9^	0.976 (0.970 – 0.983)	1.16 × 10^−12^
Sex				
Female	–	–	–	–
Male	1.64 (1.50 – 1.80)	1.69 × 10^−27^	1.77 (1.54 – 2.04)	1.50 × 10^−15^
Ethnicity				
White	–	–	–	–
Afro-Caribbean	0.932 (0.635 – 1.31)	0.702	0.968 (0.653 – 1.38)	0.866
Asian	0.720 (0.500 – 9.99)	0.0621	0.705 (0.484 – 0.992)	0.0555
Mixed	0.847 (0.424 – 1.49)	0.601	0.865 (0.430 – 1.53)	0.650
Other	0.493 (0.237 – 0.892)	0.0346	0.477 (0.277 – 0.871)	0.0288
Townsend Deprivation Index	1.06 (1.05 – 1.07)	1.17 × 10^−17^	1.03 (1.01 – 1.04)	1.86 × 10^−4^
Smoking				
Never	–	–	–	–
Current	2.51 (2.22 – 2.84)	1.85 × 10^−49^	2.08 (1.83 – 2.37)	8.01 × 10^−29^
Previous	1.59 (1.44 – 1.75)	3.22 × 10^−20^	1.30 (1.17 – 1.44)	8.06 × 10^−7^
Alcohol intake				
Never	1.29 (1.09 – 1.51)	2.14 × 10^−3^	1.12 (0.942 – 1.33)	0.197
Special occasions only	1.13 (0.971 – 1.31)	0.110	1.02 (0.874 – 1.19)	0.777
1 – 3 times per month	0.944 (0.803 – 1.11)	0.498	0.939 (0.796 – 1.11)	0.455
1 – 2 times per week	–	–	–	–
3 – 4 times per week	0.871 (0.763 – 0.993)	0.0393	0.901 (0.787 – 1.03)	0.126
Daily or almost daily	0.983 (0.861 – 1.12)	0.799	0.927 (0.807 – 1.06)	0.280
Anthropometric factors				
Height	1.13 (1.08 – 1.18)	3.43 × 10^−8^	1.08 (1.02 – 1.16)	0.0140
BMI (kg/m^2^)	1.41 (1.36 – 1.46)	1.53 × 10^−72^	1.13 (1.08 – 1.19)	2.23 × 10^−7^
Underweight (<18.5)	1.90 (1.01 – 3.22)	0.0287	2.01 (1.06 – 3.43)	0.0189
Normal (⩾18.5–25)	–	–	–	–
Overweight (⩾25–30)	1.54 (1.36 – 1.74)	4.13 × 10^−12^	1.14 (1.01 – 1.30)	0.0388
Obese (>30)	2.67 (2.37 – 3.02)	1.24 × 10^−56^	1.46 (1.27 – 1.68)	1.26 × 10^−7^
Plasma lipids				
Triglycerides	1.18 (1.14 – 1.22)	8.20 × 10^−21^	0.999 (0.953 – 1.05)	0.975
LDL	0.949 (0.901 – 0.998)	0.0435	1.05 (0.995 – 1.11)	0.0723
HDL	0.525 (0.462 – 0.595)	2.05 × 10^−23^	1.10 (0.939 – 1.29)	0.228
Glycaemic factors				
HbA1c	1.26 (1.21 – 1.31)	1.63 × 10^−30^	1.00 (0.936 – 1.07)	0.974
Diabetes mellitus without complications	2.16 (1.91 – 2.44)	1.41 × 10^−35^	1.25 (1.08 – 1.45)	3.23 × 10^−3^
Diabetes mellitus with complications	3.93 (3.23 – 4.73)	6.81 × 10^−45^	1.80 (1.40 – 2.30)	3.05 × 10^−6^
Comorbidities				
Hypertension	2.08 (1.90 – 2.28)	1.15 × 10^−57^	1.37 (1.24 – 1.52)	1.41 × 10^−9^
Carpal tunnel syndrome	12.8 (11.7 – 14.0)	<2.23 × 10^−308^	11.7 (10.6 – 12.9)	<2.23 × 10^−308^
Osteoarthritis	3.24 (2.97 – 3.54)	1.51 × 10^−149^	2.12 (1.92 – 2.34)	2.97 × 10^−49^
Rheumatoid arthritis	3.00 (2.49 – 3.58)	2.09 × 10^−32^	1.72 (1.42 – 2.07)	2.07 × 10^−8^
Hypothyroidism	1.40 (1.21 – 1.61)	3.51 × 10^−6^	1.20 (1.03 – 1.39)	0.0177
GORD	2.31 (2.09 – 2.54)	2.02 × 10^−64^	1.66 (1.50 – 1.83)	1.01 × 10^−22^

Univariable analysis – no adjustments made. Fully adjusted analysis – adjusted for age, sex, ethnicity, Townsend deprivation index, height, BMI, triglycerides, LDL cholesterol, HDL cholesterol, HbA1c, alcohol, smoking, diabetes mellitus with and without complications, hypertension, carpal tunnel syndrome, rheumatoid arthritis, hypothyroidism and GORD. Total cholesterol excluded as colinear with HDL and LDL cholesterol. Osteoarthritis excluded as it was found to act as a mediator between age and CuTS. For all: *n* = 400,610, controls = 398,639, cases = 1971.

Scaling: HbA1c centred to 42 mmol/mol and scaled so each unit increase in OR corresponds to a 10 mmol/mol increase in HbA1c; height scaled by standard deviation of overall cohort (9.28 cm) so that each unit increase in OR corresponds to additional 9.28 cm in height; BMI scaled by 5 kg/m^2^ so that each unit increase in OR corresponds to an additional 5 kg/m^2^ in BMI.

Bonferroni correction applied. Significance threshold *p* < 1.85 × 10^−3^ (27 comparisons).

OR: odds ratio.

Age was not associated with CuTS after full adjustment (OR 0.991, 95% CI: 0.984–0.997, *p* = 2.97 × 10^−3^, Bonferroni cut-off *p* < 1.85 × 10^−3^). However, given the well-established relationship between older age and osteoarthritis (Loeser et al., 2016), this relationship was examined more closely. Inclusion of osteoarthritis in the fully adjusted model caused the *p*-value for the age–CuTS association to change by six orders of magnitude (10^−3^ to 10^−9^), suggesting that osteoarthritis explains much of the apparent age–CuTS association (i.e. older age predisposes to osteoarthritis, which in turn predisposes to CuTS). The interaction between age and osteoarthritis was therefore examined. In participants without osteoarthritis, younger age was associated with increased CuTS risk in the univariable model (OR 0.981 per year, 95% CI: 0.975–0.987, *p* = 1.38 × 10^−9^), with approximately 2% higher odds for each year younger. In contrast, among participants with osteoarthritis, there was no significant association between age and CuTS risk (OR 1.00 per year, 95% CI: 0.996–1.01, *p* = 0.591). This interaction demonstrated that age affects CuTS risk differentially depending on osteoarthritis status, with the direct age–CuTS relationship being driven by younger individuals without osteoarthritis rather than by older individuals with osteoarthritis. Of note, relative differences in age are within the context of UKB and the age bracket (40–69 years) that was recruited.

### Social factors

Smoking was strongly associated with CuTS. Odds were double in current smokers compared with never smokers (OR 2.08, 95% CI: 1.83–2.37, *p* = 8.01 × 10^−29^) and 30% higher in previous smokers (OR 1.30, 95% CI: 1.17–1.44, *p* = 8.06 × 10^−7^). There was no significant association between alcohol intake and CuTS. Increased socioeconomic deprivation as measured by the Townsend deprivation index showed a weak association with CuTS (OR 1.03, 95% CI 1.01–1.04, *p* = 1.86 × 10^−4^).

### Anthropometric factors and plasma lipids

Increased BMI was associated with an elevated risk of CuTS. For every 5 kg/m^2^ increase in BMI (equivalent to the increment separating normal from overweight, and overweight from obese categories) there was a 13% increase in odds (OR 1.13, 95% CI: 1.08–1.19, *p* = 2.23 × 10^−7^). When stratified into underweight, normal, overweight and obese categories, both overweight and obese categories increased odds of CuTS in the univariable model (overweight: OR 1.54, 95% CI: 1.36–1.74, *p* = 4.13 × 10^−12^; obese: OR 2.67, 95% CI: 2.37–3.02, *p* = 1.24 × 10^−56^). However, the association only remained for the obese category in the fully adjusted model (OR 1.46 95% CI: 1.27–1.68, *p* = 1.26 × 10^−7^). Height, used as a proxy for skeletal differences in cubital tunnel morphology, showed no association with CuTS (OR 1.08, 95% CI: 1.02–1.16, *p* = 0.014).

In the univariable model, higher HDL was associated with a reduced risk of CuTS (OR 0.525, 95% CI: 0.462–0.595, *p* = 2.05 × 10^−23^), whereas elevated triglycerides were associated with increased risk (OR 1.18, 95% CI: 1.14–1.22, *p* = 8.20 × 10^−21^). However, after full adjustment, these relationships were completely abrogated. LDL levels did not show an association in either model.

### Comorbidities

Our analysis revealed significant associations between CuTS and multiple comorbidities. Even after full adjustment, CTS was associated with an 11-fold increase in CuTS odds (OR 11.7, 95% CI: 10.6–12.9, *p* < 2.23 × 10^−308^). Odds for CuTS were also increased by 37% in hypertensive individuals (OR 1.37, 95% CI: 1.24–1.52, *p* = 1.41 × 10^−9^), by 72% in individuals with RA (OR 1.72, 95% CI: 1.42–2.07, *p* = 2.07 × 10^−8^) and by 66% in individuals with GORD (OR 1.66, 95% CI: 1.50–1.83, *p* = 1.01 × 10^−22^). While osteoarthritis was excluded from the multivariable model in the main analysis owing to its complex relationship with age, its re-inclusion specifically to assess its independent effect on CuTS risk showed that it increased odds by over 2-fold (OR 2.12, 95% CI: 1.92–2.34, *p* = 2.97 × 10^−49^).

Individuals with diabetes with microvascular complications (neuropathy, nephropathy or retinopathy) had 80% increased odds of CuTS compared to non-diabetics (OR 1.80, 95% CI: 1.40–2.30, *p* = 3.05 × 10^−6^). However, after full adjustment, neither uncomplicated diabetes (OR 1.25, 95% CI: 1.08–1.45, *p* = 3.23 × 10^−3^) nor increased HbA1c (OR 1.00, 95% CI: 0.936–1.07, *p* = 0.974) showed a significant association with CuTS. Similarly, hypothyroidism did not show an association with CuTS (OR 1.20, 95% CI: 1.03–1.39, *p* = 0.02).

A sensitivity analysis was performed using a more restrictive definition of CuTS and this had minimal effect on the strength of associations found in the main analysis (Tables S3 and S7).

## Discussion

To address the considerable uncertainty on CuTS risk factors in the literature, we leveraged a population-based cohort of over 500,000 individuals and carefully corrected for covariates in our multivariable regression model. Our results verify the two established associations of smoking and CTS, clarify the relationship with male sex, age, BMI and diabetes, and substantiate poorly studied associations of hypertension, osteoarthritis, RA and GORD.

Modifiable risk factors associated with CuTS in our study were smoking, diabetes with complications, increased BMI and hypertension. The odds of CuTS were 30% and 108% higher in previous and current smokers compared with never-smokers, respectively. This aligns with previous reports of a dose-related increase in CuTS risk and decrease in ulnar nerve function with pack-years of smoking (Frost et al., 2013; Mondelli et al., 2020; Richardson et al., 2009). Previous reports have both supported (Rydberg et al., 2020) and refuted (Rocks et al., 2024) diabetes as a risk factor for CuTS; our results demonstrate 80% increased odds of CuTS in individuals with complicated diabetes, and that odds increase with progression in severity from uncomplicated to complicated diabetes. The relationship between BMI and CuTS risk has been particularly inconsistent in smaller studies (Bartels and Verbeek, 2007; Frost et al., 2013; Mondelli et al., 2020; Richardson et al., 2001; Rydberg et al., 2020). Our work in a substantially larger cohort shows that a 5 kg/m^2^ increase in BMI increases odds by 13% despite correction for diabetes, hypertension and hyperlipidaemia. Finally, hypertensive individuals had 77% increased odds of CuTS.

The foregoing modifiable risk factors may be linked by their propensity to induce a hypoxic/ischaemic and pro-inflammatory environment around the ulnar nerve. The effects of diabetes (Feldman et al., 2019), obesity (O’Brien et al., 2017), smoking (Feldman et al., 2019; Rodriguez-Fontan et al., 2020) and hypertension (Feldman et al., 2019) on either causing or exacerbating existing peripheral neuropathy are well established. In CuTS, the restrictive anatomy of the cubital tunnel provides the initial ischaemic insult of mechanical compression, which itself causes intraneural ischaemia, oedema and fibrosis (Burahee et al., 2021; Schmid et al., 2020), that may then be exacerbated by a hostile nerve milieu. Consequently, counselling patients about the association of smoking, poorly controlled diabetes, high BMI and hypertension with CuTS, and the potential merits of addressing these risk factors, may be beneficial.

Considering non-modifiable risk factors, our analysis confirmed (Mondelli et al., 2020; Richardson et al., 2001) male sex as a risk factor for CuTS. We uncovered an interesting divergence in the effects of age on CuTS, with: (1) an effect that osteoarthritis mediates (older age predisposing to osteoarthritis, which consequently predisposes to CuTS); and (2) a direct effect in which younger age (relative to the UKB cohort of 40–69 years) elevates CuTS risk. In those without osteoarthritis, younger age was subtly but significantly associated with CuTS. Odds were 2% higher for each year younger, conceivably related to increased upper limb usage and thereby dynamic ulnar nerve compression during flexion movements (James et al., 2011). In those with osteoarthritis, age did not have a significant direct effect on CuTS risk. Osteoarthritis, however, more than doubled the odds of CuTS. Osteoarthritis is well established as a disease of older age (Loeser et al., 2016) and therefore probably mediates much of the risk of older age on CuTS. Mechanistically, this may be due to increased medial osteophyte presence (Kawanishi et al., 2014) and elevated cubital tunnel pressure during flexion (Iba et al., 2008) in osteoarthritic elbows afflicted by CuTS. In analysing the interaction effects between age and osteoarthritis, our results reconcile conflicting reports of older (Grisdela Jr et al., 2025) and younger age (Rocks et al., 2024) conferring CuTS risk.

We confirm (Johnson et al., 2021b) an extremely strong association between CTS and CuTS (OR 11.7, 95% CI: 10.6–12.9, *p* < 2.23 × 10^−308^). Genome-wide association studies have shown that genetic susceptibility to CTS resides largely in the aberrant expression of extracellular matrix genes in the connective tissue environment of the median nerve, including the paraneurium (the layer directly surrounding peripheral nerves, known in the carpal tunnel as the sub-synovial connective tissue) (Patel et al., 2022; Skuladottir et al., 2022; Wiberg et al., 2019). This potentially predisposes to paraneurial fibrosis, nerve tethering and consequently ischaemia of the encased median nerve with wrist motion (Wiberg et al., 2019, 2024). We hypothesize an ‘entrapment neuropathy phenotype’ to explain the CTS–CuTS overlap: certain individuals are genetically predisposed to compression neuropathies through paraneurial fibrosis in response to mechanical and inflammatory insults to peripheral nerves. Despite this overlap, distinct differences remain – most notably, the strong female preponderance in CTS (Wiberg et al., 2021) and male predominance in CuTS. This suggests a complex interplay between hormonal influences and the genetic, metabolic and mechanical factors in the pathophysiology of the two conditions.

We demonstrate a robust association between CuTS and RA. Rheumatoid arthritis could plausibly contribute to ulnar neuropathy at the elbow by: inflammation of the elbow joint or olecranon bursa; elbow joint subluxation or valgus deformity; or mononeuritis multiplex (DeQuattro and Imboden, 2017). Rheumatoid arthritis is a well-known risk factor for CTS (Wiberg et al., 2021), but our model, in correcting for CTS, suggests that RA is independently associated with CuTS. Finally, we report a novel association between CuTS and GORD that persists despite correction for BMI. While seemingly biologically unrelated, they may be linked by non-steroidal anti-inflammatory drug use to manage pain in CuTS.

Our findings should be interpreted with the limitations of this study in mind: its cross-sectional nature preventing directionality of associations being inferred; the lack of electrodiagnostic/ultrasonographic verification of CuTS diagnosis; and the lack of biomechanical or anatomical variables. Selection biases affecting the UKB must also be considered: the cohort is healthier and less socioeconomically deprived (Fry et al., 2017) (the ‘healthy volunteer’ bias); it predominantly includes white British and urban-dwelling individuals; and it is marginally enriched for females (54% vs. 51% in the overall UK population). Despite this leading to less accurate prevalence estimates, the UKB still provides a valid setting to study exposure–disease associations (Fry et al., 2017). These limitations are balanced by the study’s notable strengths: the statistical power conferred by the sample size; the use of hospital/GP codes for phenotyping; its population- rather than a hospital-based cohort allowing greater generalizability; and our stringent statistical adjustment for testing multiple exposure phenotypes through a Bonferroni correction.

This large and comprehensive population-based study of biological risk factors for CuTS suggests that CuTS is not just a case of anatomical nerve compression, but a pathology exacerbated by systemic risk factors that promote an inflammatory and ischaemic ulnar nerve environment. As many of these risk factors are modifiable, our work provides impetus for a preventative approach to the condition.

## Supplemental Material

sj-docx-1-jhs-10.1177_17531934261426700 – Supplemental material for The epidemiology of cubital tunnel syndrome: a UK Biobank case–control study Supplemental material, sj-docx-1-jhs-10.1177_17531934261426700 for The epidemiology of cubital tunnel syndrome: a UK Biobank case–control study by Maria Lucey, Frederik Heymann Lassen, Dominic Furniss and Akira Wiberg in Journal of Hand Surgery (European Volume)
