# Supplementary material for: The epidemiology of cubital tunnel syndrome: a UK Biobank case–control study
Source: J Hand Surg Eur Vol. 2026 Mar 11;51(8):992–1002. doi: 10.1177/17531934261426700 (PMC13490649; doi:10.1177/17531934261426700)
Supplement: Supplementary file 1 [file sj-docx-1-jhs-10.1177_17531934261426700.docx]

**Supplementary Content**

**Table of Contents**

[Table S1: Diagnostic Codes Used to Identify Outcome and Exposures	2](#_Toc215753752)

[Table S2: Read Codes (CTV3) Used to Identify Exposures and Outcomes	4](#_Toc215753753)

[Table S3: Cubital Tunnel Syndrome Definitions	7](#_Toc215753754)

[Supplementary Note 1: Missingness Analysis Supporting Explanation	8](#_Toc215753755)

[Table S4: Missingness for Variables and Fisher Exact Test *P*-values	10](#_Toc215753756)

[Table S5: *P*-values for Varying Delta Values in the Fully-Adjusted Model	11](#_Toc215753757)

[Table S6: Phenome-Wide Association Study Top 15 Associations Based on *P*-value	12](#_Toc215753758)

[Table S7: Univariable and Fully-Adjusted Logistic Regression ORs for Cubital Tunnel Syndrome Using Restrictive Definition	13](#_Toc215753759)

# Table S1. Diagnostic codes used to identify outcome and exposures.

| **Exposure** | **UK Biobank Data Fields** | **OPCS-4 Code^1^** | **ICD-10 Code^2^** | **ICD-9 Code^3^** | **Non-Cancer Illness Code^4^** | **Operation Code^5^** |
| --- | --- | --- | --- | --- | --- | --- |
| Cubital Tunnel Syndrome |  | A67.1 | G56.2 | 3542 |  |  |
| Age | 34 |  |  |  |  |  |
| Sex | 22001 |  |  |  |  |  |
| Ethnicity | 21000 |  |  |  |  |  |
| Height | 50 |  |  |  |  |  |
| BMI | 21001 |  |  |  |  |  |
| Diabetes Mellitus* |  |  | E10, E11, E13, E14, T38.3, Y42.3, O24.0, O24.1, O24.3 | 250, 250.0, 250.1, 250.2 | 1220, 1221, 1222, 1223 |  |
| Diabetes Mellitus with Complications* |  |  | E10.2, E10.3, E10.4, E10.5, E10.6, E10.7, E10.8, E11.2, E11.3, E11.4, E11.5, E11.6, E11.7, E11.8, E13.2, E13.3, E13.4,  E13.5, E13.6, E13.7, E13.8, E14.2, E14.3, E14.4, E14.5, E14.6, E14.7, E14.8, G59.0, G63.2, H28.0, H36.0, M14.2, N08.3 | 250.3, 250.4, 250.5, 250.6, 362.0 | 1607, 1276, 1468 |  |
| Total Cholesterol | 30690 |  | - | - | - | - |
| Triglycerides | 30870 |  |  |  |  |  |
| HDL Cholesterol | 30760 |  |  |  |  |  |
| LDL Cholesterol | 30780 |  |  |  |  |  |
| HbA1c | 30750 |  |  |  |  |  |
| Alcohol Intake | 1558 |  |  |  |  |  |
| Smoking | 20116 |  |  |  |  |  |
| Hypertension |  |  | I10., I11., I12., I13., I15. | 401., 402., 403., 404., 405. | 1065, 1072 | - |
| Townsend Deprivation Index | 22189 | - | - | - | - | - |
| Carpal Tunnel Syndrome |  | A651, A692 | G56.0 | 354.0 | 1541 | 1501 |
| Osteoarthritis |  |  | M15, M16, M17, M18, M19, M257 | 7151, 7153 | 1465 | - |
| Rheumatoid Arthritis |  |  | M05, M06 | 7140, 7141, 7142 | 1464 | - |
| Hypothyroidism |  |  | E02, E03 | 2449 | 1226 | - |
| GORD |  |  | K21 | 5301 | 1138 | - |

* DM without complications = [DM cases] minus [DM with complications cases]

DM with complications includes microvascular (e.g. neuropathy, retinopathy, nephropathy) complications

GORD: gastroesophageal reflux disease

^1^ OPCS code UK Biobank Data-Field: 41272

^2^ ICD-10 code UK Biobank Data-Field: 41270

^3^ ICD-9 code UK Biobank Data-Field: 41271

^4^ Non-cancer illness code: Self-report, UK Biobank Data-field: 20002

^5^ Operation code: Self-report, UK Biobank Data-field: 20004

# Table S2. Read Codes (CTV3) used to identify exposures and outcomes.

| **Exposure** | **CTV3 Codes** |
| --- | --- |
| Cubital Tunnel Syndrome | F3420 – Cubital tunnel syndrome  70580 – Decompression of ulnar nerve at elbow  X00Br – Ulnar nerve entrapment at elbow |
| Age |  |
| Sex |  |
| Ethnicity |  |
| Height |  |
| BMI |  |
| Diabetes Mellitus | 66A3, 66A4, 66A5, C10.., C100., C1000, C1001, C100z, C101., C1010, C1011, C101y, C101z, C102., C1020, C1021, C102z, C103., C1030, C1031, C103y, C103z, C108., C1084, C1088, C1089, C108A, C108E, C109., C1097, C1099, C109D, C109J, C109K, C10A., C10A0, C10A1, C10C., C10D., C10E., C10E4, C10E8, C10E9, C10EA, C10EE, C10EM, C10EN, C10EQ, C10ER, C10F., C10F7, C10F9, C10FD, C10FJ, C10FK, C10FN, C10FP, C10FQ, C10FR, C10FS, C10G., C10G0, C10M., C10N., C10N0, C10N1, L1805, L1806, L180X, Lyu29, X40J4, X40J5, X40J6, X40J7, X40JA, X40JB, X40JI, X40JJ, XE10E, XE10F, XSETe, Xa4g7, XaELP, XaELQ, XaFWG, XaFWI, XaIrf, XaJlL, XaJlR, XaKyW, XaKyX, XaMzI, XaOPt, XaOPu |
| Diabetes Mellitus with Complications* | C104, C1040, C1041, C104y, C104z, C105., C1050, C1051, C105y, C105z, C106., C1060, C1061, C106y, C106z, C107., C1070, C1071, C1072, C1073, C1074, C107z, C1080, C1081, C1082, C1083, C1085, C1086, C1087, C108B, C108C, C108D, C108F, C108G, C108H, C108J, C108y, C108z, C1090, C1091, C1092, C1093, C1094, C1095, C1096, C109A, C109B, C109C, C109E, C109F, C109G, C109H, C10A5, C10E0, C10E1, C10E2, C10E3, C10E5, C10E6, C10E7, C10EB, C10EC, C10ED, C10EF, C10EG, C10EH, C10EJ, C10EK, C10EL, C10EP, C10F0, C10F1, C10F2, C10F3, C10F4, C10F5, C10F6, C10FA, C10FB, C10FC, C10FE, C10FF, C10FG, C10FH, C10FL, C10FM, C10y., C10y1, C10yy, C10yz, C10z., C10z0, C10z1, C10zy, C10zz, X00Ag, X30Kk, XE10G, XE10H, XE10I, XE15k, XM1Qx, Xa0lK, XaEnn, XaEno, XaEnp, XaEnq, XaF04, XaF05, XaFm8, XaFmA, XaFmK, XaFmL, XaFmM, XaFn7, XaFn8, XaFn9, XaIzM, XaIzN, XaIzQ, XaIzR, XaJQp, XaJSr, XaPmX |
| Total Cholesterol |  |
| Triglycerides |  |
| HDL Cholesterol |  |
| HbA1c |  |
| Alcohol Intake |  |
| Smoking |  |
| Hypertension | 14A2,1JD., 21261, 212K., 246M., 662..12, 6624, 6627, 6628, 6629, 662b., 662c., 662d., 662F., 662G., 662H., 662O., 662P., 662P0, 662q., 662r., 67H8., 7Q01., 7Q01y, 8B26., 8BL0., 8CR4., 8HT5., 8I3N., 9h3.., 9h31., 9h32., 9N03., 9N1y2, 9N4L., 9OI.., 9OI..11, 9OI1., 9OI2., 9OI3., 9OI4., 9OI5., 9OI6., 9OI7., 9OI8., 9OI9., 9OIA., 9OIA.11, 9OIZ., F4042, F4213, G2..., G2...11, G20.., G20..11, G20..12, G200., G201., G202., G203., G20z., G20z.11, G21.., G210., G2100, G2101, G210z, G211., G2110, G2111, G211z, G21z., G21z0, G21z011, G21z1, G21zz, G22.., G22..11, G220., G221., G222., G22z., G22z.11, G23.., G230., G231., G232., G233., G234., G23z., G24.., G240., G2400, G240z, G241., G2410, G241z, G244., G24z., G24z0, G24z1, G24zz, G25.., G25..11, G26.., G26..11, G27.., G28.., G2y.., G2z.., G672., G672.11, Gyu2., Gyu20, Gyu21, L122., L1220, L1221, L1223, L122z, L127., L127z, L128., L1280, L1282, TJC7., TJC7z, U60C5, U60C51, U60C51A, XE0Ub, XaZzo, XaZbz, XaZWn, XaZWm, Xab9M, Xab9L, XE0Uc, XE0Ud, Xa0kX, 61462, Xa3fQ |
| Townsend Deprivation Index |  |
| Carpal Tunnel Syndrome | XE15g – Carpal tunnel syndrome  70560 – Decompression of median nerve at wrist  70564 – Endoscopic carpal tunnel release  70562 – Re-release of carpal tunnel  XaPuG – Revision of carpal tunnel release  XaPpH – Revision of decompression of median nerve at wrist |
| Osteoarthritis | N0501, N114., N0535, N05z., Nyu23, X7031, X76G5, N0503, XaLsk, X704R, N0506, XaIna, X7035, X7030, N050., N050z, N0502, XE1DW, N0500, 14G2., XM05w, N0507, N0504, X7038, X7041, N053., N053z, N0538, N0531, N0537, N0533, N0534, XE1DZ, N0532, N0530, XE1DY, N051., N051z, XaEGf, N0518, N0517, N0513, N0514, N0516, N0515, N0511, N0512, XaEGd, XaEGe, N0510, N052., N052z, N0528, XE1DX, N0527, N0523, N0524, N0526, N0521, N0522, N0520, 2G26., XaBzh, N0540, N054., N0549, N0547, N0543, N0544, N0546, N0545, N0541, N0542, N0548, XE1DV, N05.., Xa3gR, Xa3gQ, Xa3gS, XE1Gm, N05zz, XE1Da, N05zS, N05zB, N05zN, XE1Dg, N05zH, N05zD, N05zC, N05zJ, N05zU, N05zL, N05zT, N05zF, N05zR, N05zG, N05zK, N05z9, N05z1, N05zA, N05zP, N05zQ, XE1Dc, XE1Dd, XE1Df, XE1Db, N05zM, N05z0, N05zE, N05z8, XE1De, N05z7, N05z4, N05z6, N05z3, N05z2, N05z5, X703C, X703M, X703H, X703D, X703G, X703F, X703O, XaBmY, X703K, X703L, XM1NQ, X703J, N054z, X703I, X703B, X703A, X703N, X703P, X703E, N0536, X7042, XaYQD, N0529, N0519, X703Q, X7034, X702z |
| Rheumatoid Arthritis | 14G1., 2G27, F3712, F3964, G5y8., G5yA., N040., N0400, N0401, N0402, N0403, N0404, N0405, N0406, N0407, N0408, N0409, N040A, N040B, N040C, N040D, N040E, N040F, N040G, N040H, N040J, N040K, N040L, N040M, N040Q, N040S, N040T, N041., N042., N0421, N0422, N042z, N04X., Nyu10, Nyu11, Nyu12, Nyu1G, X103Y, X103Y, X701h, X701i, X701j, X701k, X701k, X701l, X701m, X705t, X705u, X705v, Xa3gL, Xa3gM, Xa3gN, Xa3gO, Xa3gP, XaBMO, XaZdB, XE1DU, XM1XV |
| Hypothyroidism | X40IQ, XE108, X40HI, X005I, C04y., X40HD, XE10A, Xa3ed, XaLUg, XaOjl, X40HD, XaJuI, XaMFm, XaKbz, XaJuO, XaJuN, XaJuM, C06y1, X40I1, F3953, F11x5, F1441, PK251, X00Ao, 1432., C04z0, C05y., C03.., Xa0l7, X40H8, PK251, X40HA, X40HB, X40HC, C03y., XE107, X40I7, X40I8, X40I9, X40HL, X40HM, XE109, C0410, X40HN, C041., C041z, C043., C043z, X40HO, C0430, C0431, C0432, X40HP, X40IB, C042., C1343, X40LB, X40LC, X40Hv, X40Hw, X40Hx, X40Hy, X40Hz, Cyu11, Xa3ec, Xa08g, Q4337 |
| Gastroesophageal Reflux Disease | X3003, 171J.00, 1953, 1953.11, 1954, 1955, 1955.11, 1956, 1957, 1958, 195Z.00, J1011, J101111, J101112, J101113, J10y400, J10y411, J10y412, J10y413 |

# Table S3. Cubital tunnel syndrome definitions.

|  | **OPCS-4** | **ICD-10** | **ICD-9** | **CTV3** |
| --- | --- | --- | --- | --- |
| **Adopted case definition** | A67.1 (“Cubital tunnel release”) | G56.2 (“Lesions of ulnar nerve”) | 3542 (“Lesions of ulnar nerve”) | F3420 (“Cubital tunnel syndrome”)  70580 (“Decompression of ulnar nerve at elbow”)  X00Br (“Ulnar nerve entrapment at elbow”) |
| Contributing cases: | 1,458 | 2,110 | 12 | 515 |
| Overall cases^1^: | 2,487 | | | |
| Complete Case Analysis^2^: | 1,971 | | | |
| **Restrictive case definition** | A67.1 (“Cubital tunnel release”) | G56.2 (“Lesions of ulnar nerve”) AND OPCS-4 Z81.5 (“Elbow joint”)* | 3542 (“Lesions of ulnar nerve”) AND OPCS-4 Z81.5 (“Elbow joint”) | F3420 (“Cubital tunnel syndrome”)  70580 (“Decompression of ulnar nerve at elbow”)  X00Br (“Ulnar nerve entrapment at elbow”) |
| Contributing cases: | 1,458 | 161 | 0 | 515 |
| Overall cases^1^: | 1,829 | | | |
| Complete Case Analysis^2^: | 1,444 | | | |

**^1^** Overall cases = intersection of individual categories i.e. accounting for individuals who may have two separate diagnostic codes.

^2^ Complete Case Analysis (CCA) = cases remaining after removing all individuals with missing data in multivariable analysis.

* OPCS-4 code for “Elbow joint” combined with ICD-10 G56.2 to try and provide anatomical specificity. However, OPCS-4 codes for anatomical locations not commonly or consistently used in combination with other OPCS-4 codes to define the location of surgery (e.g. in the UK Biobank, there are only 8,520 counts for Z84.3 “Hip joint” whilst there are 12,104 counts for W37 “Total prosthetic replacement of the hip joint using cement”, 9,302 counts for W38 “Total prosthetic replacement of the hip joint not using cement”, and 2,160 counts for W39 “Other total prosthetic replacement of hip joint”, even before considering other hip joint surgeries). Combining this code dropped the case count significantly, and given that the restrictive definition did not notably change the results of multivariable analysis (see table S6), this definition was not used for the main analysis.

# Supplementary note 1: Missingness analysis supporting explanation

Missing data were observed across eleven of twenty tested variables: ethnicity, Townsend deprivation index, smoking, alcohol, height, BMI, total cholesterol, triglycerides, LDL cholesterol, HDL cholesterol, and HbA1c. Missingness ranged from 0.08% to 14.6%. We first tested whether missingness in these variables was related to our outcome (cubital tunnel syndrome) using Fisher’s exact test (two-sided; Table S4). This was the case for height (*P*=1.53×10^-4^) and BMI (*P*=4.19×10^-4^), which we further interrogate below.

We then applied Little’s test (R function ‘mcar_test’ of ‘naniar’ library) to determine whether missing data followed a Missing Completely at Random (MCAR) pattern. The test rejected the null hypothesis, ruling out MCAR as the underlying missingness mechanism and indicating that the missing data were either Missing at Random (MAR) or Missing Not at Random (MNAR).

Multiple imputation requires data to be MAR to produce unbiased results. This method allows analysis of records with missing information by generating plausible values based on observed data patterns. However, if data are MNAR – where missingness relates to the unobserved values themselves – imputation can introduce bias. For instance, individuals with higher BMI might decline weight measurement due to obesity-related stigma, creating systematic patterns in missing data. Given this possibility, we could not rule out MNAR mechanisms, making complete imputation potentially problematic for our primary analysis.

When data are MNAR but missingness is unrelated to the outcome, complete case analysis (CCA) often provides less biased estimates than multiple imputation (Bartlett et al., 2015; Hughes et al., 2019). We therefore conducted our main analysis using CCA with 400,610 individuals (398,639 controls and 1,971 cubital tunnel cases).

Since missingness in height and BMI was associated with our outcome, we performed sensitivity analyses using Multiple Imputation by Chained Equations (MICE) (Buuren and Groothuis-Oudshoorn, 2011). Missing records were imputed for height and BMI sampling from distributions with a mean zero, one, or two standard deviations from the measured population mean (Table S5). Height showed no association with cubital tunnel syndrome across most scenarios, with borderline significance only when imputing values two standard deviations above the mean (*P*=1.22×10^-3^) — this represents a highly implausible scenario for all missing data. The association between increased BMI and cubital tunnel syndrome remained significant even when imputing values from two standard deviations above (*P*=1.26×10^-6^) or below (*P*=3.48×10^-5^) the population mean. Together, these analyses support the robustness of our findings despite missing data patterns.

References:

Bartlett JW, Harel O, Carpenter JR. Asymptotically unbiased estimation of exposure odds ratios in complete records logistic regression. Am J Epidemiol. 2015, 182: 730–6.

Buuren S van, Groothuis-Oudshoorn K. mice : Multivariate imputation by chained equations in R. J Stat Softw. 2011, 45.

Hughes RA, Heron J, Sterne JAC, Tilling K. Accounting for missing data in statistical analyses: multiple imputation is not always the answer. Int J Epidemiol. 2019, 48: 1294–304.

# Table S4. Missingness for variables and Fisher Exact Test *p*-values

| **Exposure** | **Missing controls (%)** | **Missing cases (%)** | ***P*-value** |
| --- | --- | --- | --- |
| Ethnicity | 2,760 (0.55) | 15 (0.60) | 0.684 |
| Townsend Deprivation Index | 622 (0.12) | 2 (0.08) | 0.776 |
| Smoking | 2,936 (0.59) | 12 (0.48) | 0.598 |
| Alcohol | 1,494 (0.30) | 6 (0.24) | 0.852 |
| Height | 2,509 (0.50) | 28 (1.1) | 1.53×10^-4^ |
| BMI | 3,072 (0.61) | 31 (1.3) | 4.19×10^-4^ |
| Total Cholesterol | 32,720 (6.6) | 165 (6.6) | 0.839 |
| Triglycerides | 33,092 (6.6) | 168 (6.8) | 0.777 |
| LDL Cholesterol | 33,594 (6.7) | 173 (7.0) | 0.630 |
| HDL Cholesterol | 72,212 (14.5) | 362 (14.6) | 0.886 |
| HbA1c | 35,794 (7.2) | 172 (6.9) | 0.668 |

Counts for missing number of cases and controls for each variable with missing data. Two-sided Fisher’s exact test, significance threshold *P*<4.60×10^-3^ (11 tests), Bonferroni correction. Total number of controls: 499,641. Total number of cubital tunnel cases: 2,487.

# Table S5. *P*-values for varying delta values in the fully-adjusted model.

| **SD Deviation** | **BMI Delta** | **BMI *P*-value** | **Height Delta** | **Height *P*-value** |
| --- | --- | --- | --- | --- |
| *CCA* | – | 2.23×10^-7^ | – | 0.0140 |
|  |  |  |  |  |
| +2 | 9.61 | 1.26×10^-6^ | 18.6 | 1.22×10^-3^ |
| +1 | 4.80 | 2.18×10^-6^ | 9.28 | 2.65×10^-3^ |
| 0 | 0 | 4.68×10^-6^ | 0 | 6.37×10^-3^ |
| -1 | -4.80 | 1.20×10^-5^ | -9.28 | 0.0159 |
| -2 | -9.61 | 3.48×10^-5^ | -18.6 | 0.0386 |

*P*-values reported are averages from five separate imputed data sets. Delta values are the standard deviation (SD) of the population multiplied by the desired SD shift (± 1 or ± 2). Multivariable model from main analysis (with correction for same variables) used to determine *P*-values. Significance threshold *P*<1.85×10^-3^ (27 comparisons), Bonferroni correction.

# Table S6. Phenome-wide association study top 15 associations based on *p*-value.

| **ICD-10 Code** | **No. cases with ICD-10 Code** | **ICD-10 Diagnosis** | **ICD-10 Grouping** | **Fisher's test *p*-value** |
| --- | --- | --- | --- | --- |
| G560 | 16550 | Carpal tunnel syndrome | Neurological | <2.23×10^-308^ |
| M199 | 49292 | Arthrosis, unspecified | Musculoskeletal | 3.62×10^-175^ |
| I10 | 162237 | Essential (primary) hypertension | Circulatory | 4.39×10^-114^ |
| E669 | 37997 | Obesity, unspecified | Endocrine or metabolic | 1.28×10^-106^ |
| M478 | 14361 | Other spondylosis | Musculoskeletal | 4.83×10^-96^ |
| M139 | 33443 | Arthritis, unspecified | Musculoskeletal | 3.09×10^-94^ |
| M255 | 18416 | Pain in joint | Musculoskeletal | 4.97×10^-84^ |
| E119 | 43719 | Type 2 diabetes mellitus | Endocrine or metabolic | 1.80×10^-78^ |
| F329 | 32610 | Depressive episode, unspecified | Mental disorders | 1.59×10^-75^ |
| K219 | 45847 | Gastro-oesophageal reflux disease without oesophagitis | Digestive | 1.78×10^-72^ |
| M159 | 12192 | Polyarthrosis, unspecified | Musculoskeletal | 6.50×10^-71^ |
| M179 | 37052 | Gonarthrosis, unspecified | Musculoskeletal | 1.52×10^-70^ |
| E780 | 77322 | Pure hypercholesterolaemia | Endocrine or metabolic | 2.45×10^-70^ |
| F171 | 26148 | Mental and behavioural disorders due to use of tobacco | Mental disorders | 1.81×10^-67^ |
| J449 | 21286 | Chronic obstructive pulmonary disease, unspecified | Respiratory | 4.98×10^-66^ |

# Table S7. Univariable and fully-adjusted logistic regression ORs for cubital tunnel syndrome using restrictive definition.

| **Exposure** | **Univariable OR (95% CI)** | **P-value** | **Fully-Adjusted Multivariable OR (95% CI)** | **P-value** |
| --- | --- | --- | --- | --- |
| **Age** | 1.01 (0.999 – 1.01) | 0.106 | 0.998 (0.980 – 0.995) | 7.22×10^-4^ |
| **Sex** |  |  |  |  |
| Female | – | – | – | – |
| Male | 1.68 (1.51 – 1.86) | 4.58×10^-22^ | 1.79 (1.52 – 2.12) | 3.33×10^-12^ |
| **Ethnicity** |  |  |  |  |
| White | – | – | – | – |
| Afro-Caribbean | 1.02 (0.663 – 1.49) | 0.920 | 1.10 (0.704 – 1.63) | 0.659 |
| Asian | 0.777 (0.514 – 1.12) | 0.203 | 0.775 (0.505 – 1.14) | 0.216 |
| Mixed | 0.812 (0.348 – 1.58) | 0.582 | 0.843 (0.359 – 1.65) | 0.655 |
| Other | 0.526 (0.225 – 1.02) | 8.93×10^-2^ | 0.523 (0.223 – 1.03) | 0.0910 |
| **Townsend Deprivation Index** | 1.05 (1.04 – 1.07) | 4.01×10^-11^ | 1.02 (1.00 – 1.04) | 1.79×10^-2^ |
| **Smoking** |  |  |  |  |
| Never | – | – | – | – |
| Current | 2.41 (2.09 – 2.78) | 2.09×10^-33^ | 2.00 (1.72 – 2.32) | 2.29×10^-19^ |
| Previous | 1.55 (1.38 – 1.74) | 5.71×10^-14^ | 1.27 (1.13 – 1.43) | 9.76×10^-5^ |
| **Alcohol Intake** |  | | | |
| Never | 1.26 (1.04 – 1.52) | 1.54×10^-2^ | 1.09 (0.892 – 1.33) | 0.392 |
| Special occasions only | 1.10 (0.918 – 1.31) | 0.302 | 0.993 (0.826 – 1.19) | 0.937 |
| 1 – 3 times per month | 0.918 (0.758 – 1.11) | 0.378 | 0.911 (0.749 – 1.10) | 0.339 |
| 1 – 2 times per week | – | – | – | – |
| 3 – 4 times per week | 0.871 (0.747 – 1.01) | 7.53×10^-2^ | 0.905 (0.775 – 1.06) | 0.209 |
| Daily or almost daily | 0.961 (0.823 – 1.12) | 0.611 | 0.922 (0.784 – 1.08) | 0.322 |
| **Anthropometric factors** |  |  |  |  |
| Height | 1.15 (1.09 – 1.21) | 2.08×10^-7^ | 1.10 (1.02 – 1.18) | 0.0159 |
| BMI | 1.42 (1.36 – 1.48) | 3.90×10^-58^ | 1.14 (1.08 – 1.20) | 2.24×10^-6^ |
| **Plasma Lipids** |  |  |  |  |
| Triglycerides | 1.19 (1.14 – 1.24) | 2.89×10^-17^ | 1.01 (0.955 – 1.06) | 0.760 |
| LDL | 0.938 (0.883 – 0.996) | 3.64×10^-2^ | 1.04 (0.971 – 1.10) | 0.286 |
| HDL | 0.504 (0.434 – 0.584) | 1.57×10^-19^ | 1.09 (0.902 – 1.31) | 0.375 |
| **Glycaemic factors** |  |  |  |  |
| HbA1c | 1.24 (1.19 – 1.30) | 2.02×10^-20^ | 0.974 (0.900 – 1.05) | 0.505 |
| Diabetes mellitus without complications | 2.24 (1.94 – 2.57) | 2.13×10^-29^ | 1.32 (1.11 – 1.57) | 1.36×10^-3^ |
| Diabetes mellitus with complications | 4.03 (3.21 – 4.99) | 2.91×10^-35^ | 1.93 (1.44 – 2.56) | 6.81×10^-6^ |
| **Comorbidities** |  |  |  |  |
| Hypertension | 1.99 (1.79 – 2.20) | 6.37×10^-38^ | 1.28 (1.13 – 1.44) | 5.88×10^-5^ |
| Carpal tunnel syndrome | 14.3 (12.8 – 15.9) | <2.23×10^-308^ | 13.2 (11.8 – 14.7) | <2.23×10^-308^ |
| Osteoarthritis | 3.34 (3.01 – 3.70) | 2.54×10^-115^ | 2.16 (1.92 – 2.43) | 2.21×10^-38^ |
| Rheumatoid arthritis | 2.84 (2.27 – 3.50) | 3.28×10^-21^ | 1.59 (1.26 – 1.98) | 5.28×10^-5^ |
| Hypothyroidism | 1.37 (1.15 – 1.61) | 2.38×10^-4^ | 1.17 (0.979 – 1.39) | 0.0772 |
| GORD | 2.44 (2.18 – 2.72) | 5.47×10^-55^ | 1.75 (1.56 – 1.96) | 4.46×10^-21^ |

Univariable analysis – no adjustments made. Fully-adjusted analysis – adjusted for age, sex, ethnicity, Townsend deprivation index, height, BMI, triglycerides, LDL cholesterol, HDL cholesterol, HbA1c, alcohol, smoking, diabetes mellitus with and without complications, hypertension, carpal tunnel syndrome, rheumatoid arthritis, hypothyroidism, and GORD. Total cholesterol excluded as colinear with HDL and LDL cholesterol. Osteoarthritis excluded as it was found to act as a mediator between age and CuTS. For all: n = 400,610, controls = 399,166, cases = 1,444.

Scaling: HbA1c centred to 42mmol/mol and scaled so each unit increase in OR corresponds to a 10mmol/mol increase in HbA1c; height scaled by standard deviation of overall cohort (9.28cm) so that each unit increase in OR corresponds to additional 9.28cm in height; BMI scaled by 5 kg/m^2^ so that each unit increase in OR corresponds to an additional 5 kg/m^2^ in BMI.

Bonferroni correction applied. Significance threshold *P*<1.85×10^-3^ (27 comparisons).

GORD: gastroesophageal reflux disease.
